# Establishing a student-run free clinic in a major city in Northern Europe: a 1-year experience from Hamburg, Germany

**DOI:** 10.1093/pubmed/fdz165

**Published:** 2019-12-16

**Authors:** Richard Drexler, Felix Fröschle, Christopher Predel, Berit Sturm, Klara Ustorf, Louisa Lehner, Jara Janzen, Lisa Valentin, Tristan Scheer, Franziska Lehnert, Refmir Tadzic, Karl Jürgen Oldhafer, Tobias N Meyer

**Affiliations:** 1 Asklepios Campus Hamburg, Semmelweis University Budapest, Hamburg, Germany; 2 Student-Run Free Clinic Hamburg, Hamburg, Germany; 3 Gesundheitszentrum Dr. Tadzic und Kollegen, Hamburg, Germany; 4 Department of General and Visceral Surgery, Asklepios Hospital Barmbek, Hamburg, Germany; 5 Department of Nephrology, Asklepios Hospital Barmbek, Hamburg, Germany

**Keywords:** Medical education, student run clinic, SRFC, health services, health Care, medical students

## Abstract

**Background:**

Student-Run Free Clinics (SRFCs) have been an integral part of US medical schools since the 1960s and provide health care to underserved populations. In 2018, we established an SRFC in Hamburg, Germany, a major city in Northern Europe. The aim of this study was to describe the central problems and to investigate the usefulness of an SRFC in a country with free access to medical care, such as Germany.

**Methods:**

All consecutive patients treated at the SRFC Hamburg between February 2018 and March 2019 that consented to this study were analyzed regarding clinical characteristics, diagnosis, readmission rate and country of origin.

**Results:**

Between February 2018 and March 2019, 229 patients were treated at the SRFC in Hamburg. The patients came from 33 different countries with a majority (*n* = 206, 90%) from countries inside the European Union. The most common reasons for visiting the SRFC were infections (23.2%), acute or chronic wounds (13.5%) and fractures (6.3%).

**Conclusion:**

Our multicultural patients suffer mainly from infections and traumatological and dermatological diseases. We find similarities to published Canadian SRFC patient cohorts but differences in diseases and treatment modalities compared to US SRFCs. Importantly, we demonstrate the relevance and necessity of the SRFC in a major city in Northern Europe.

## Introduction

Student-Run Free Clinics (SRFCs) were developed in the USA in the 1960s and have become integral institutions in most US medical schools.^1^ Today, the number of SRFCs being established in other countries including Australia, Canada, the Netherlands, Sweden and Germany is rising.^2–6^ Regardless of the health care system of these countries, SRFCs provide basic medical treatment for underserved people who may be uninsured, homeless or have limited access to regular health care due to non-medical reasons.^7–10^ Medical students have the lead role as a provider of care for these patients and are, with support of licensed health care professionals, responsible for the operational management of the clinic. Apart from the main objective to provide health care for underserved people, SRFCs provide an innovative opportunity for interprofessional education (IPE), where students learning with, from and about each other.^10–12^ In recent studies, most students valued their experiences at SRFCs as ‘more effective than formal classroom-based instruction’ and perceived themselves with improved knowledge, skills and attitudes after volunteering in an SRFC.^11^^,^^13–16^ Furthermore, the majority of volunteering students developed empathy, heightened social awareness and are more interested to work with underserved patients after graduation.^17–20^ In contrast, non-volunteering students demonstrated declined empathy through their medical education.^21–24^ Moreover, the students have a better understanding of their role within an interprofessional team with students from other healthcare disciplines and they learn how to lead and organize a team as they take on leadership roles and individual responsibilities for incoming patients.^25^^,^^26^

In 2014, the German Federal Task Force on Homelessness reported about 39,000 homeless and 335,000 houseless persons living in Germany, but the actual numbers are suspected to be much higher.^27–31^ Even though Germany has a well-functioning healthcare system, there is evidence that a significant amount of people lack access to regular health services.^27^^,^^32–35^ Particularly, in a major city such as Hamburg, the number of people without sufficient access to health care is high and has permanently grown over the last decade.^27^^,^^29^ In our case, we established a Student-Run Free Clinic, the ‘Studentische Poliklinik’, in Hamburg, Germany. It is the second SRFC in Germany, following an SRFC established in Frankfurt in 2014.^3^

The SRFC is affiliated with a homeless shelter in the harbor district, providing free medical care by students from the Asklepios Medical School in Hamburg for 4 hours per week. The treatment team consists of three medical students from different grades, who have completed their third year of medical school. A licensed health care professional supervised and educated three students per session and made the final decision about diagnosis and treatment. Before joining the team, students are required to complete an extensive training program. All students must complete a curricular training consisting of two modules prior to working in the SRFC. The curricular training follows the principle of a peer-assisted learning program as described by Seifert *et al*. and was implemented in the curriculum of the Semmelweis University in Budapest.^3^ The primary objective of our SRFC in Hamburg is offering health care for a marginalized population while giving students the opportunity to improve clinical knowledge, social accountability and leadership skills.

The purpose of this manuscript is to share our experiences with our patient group and investigate the usefulness of an SRFC in a European country with a functioning health care system. Furthermore, we compare our data with existing programs of US and Canadian SRFCs.

## Methods

### Study design and patient cohort

We analyzed prospective data of all consecutive patients treated at the SRFC in Hamburg between February 2018 and March 2019. Patient variables included age, gender, geographical origin, first and secondary diagnoses, readmission rate, type of treatment and referral to specialists.

### Statistical analysis

Differences in continuous variables were analyzed with the Mann–Whitney U test and differences in proportions with the chi-square-test or Fisher exact test. Continuous parameters are presented as median and interquartile range (IQR). A two-sided *P* value less than 0.05 was considered as statistically significant. All analyses were performed using SPSS Inc. (Chicago, IL, USA).

### Ethical considerations

Collection and analysis of the patients’ data were processed in accordance with the guidelines of the Institutional Ethics Committee after approval (Ethics Committee Approval No. WF-027/19 Hamburg).

## Results

### Patient characteristics

Between February 2018 and March 2019, 229 patients underwent medical treatment during the weekly consultation hours at the SRFC in Hamburg. All 229 patients were available for the analysis and consented to share their data. The median age in this study population was 45 years (IQR: 36–56 years) and 41 (17.9%) out of 229 patients were female. About, 81 patients (37.7%) were treated more than once during the survey period at the SRFC. Nearly half of our patients (112 patients, 48.9%) had given information about their current work and livelihood. Only 17 patients (15.2%) had a regular income, but every patient was dependent upon welfare facilities.

### Country of origin

**Fig. 1:**
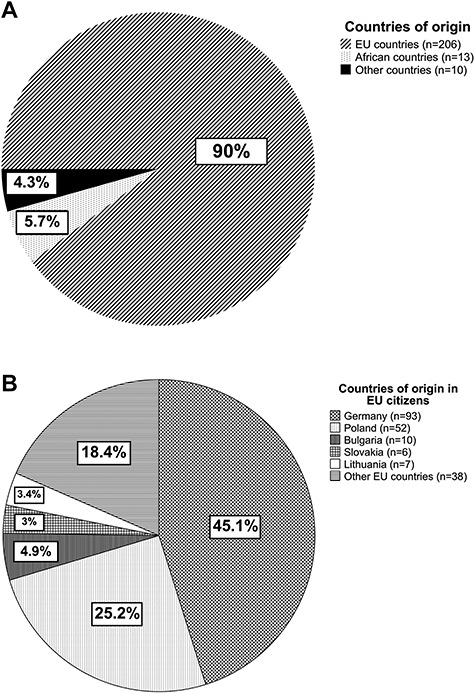
Comparison of home countries in the study population with regard to all countries (A) and European countries (B).

The population of Hamburg, Germany, is 1.8 Mio and a large international harbor contributes to a multicultural and multiethnic population. We therefore examined the nationalities of the patient cohort. Our patients came from 33 different countries worldwide but the majority (90%) were from countries within the European Union (EU, Fig. 1A and B). Of the EU citizens, 93 patients (45.1%) were German and 51.9% were from Eastern Europe.

**Fig. 2:**
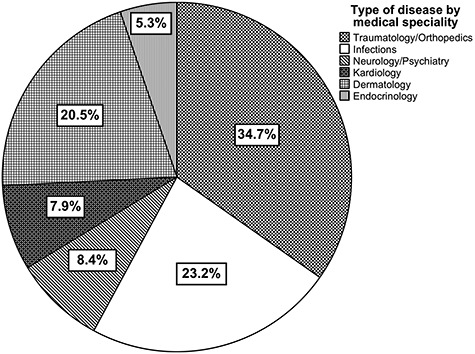
Distribution of diseases categorized by medical specialties.

Patients from Poland made up the second largest group with a percentage of 25.2%. About, 23 patients (10%) were born outside the EU and 56.5% of this group came from countries in Africa—for example Nigeria and Guinea. Of Non-EU countries represented, Barbados, Australia and Nepal were the countries furthest away.

### Diseases and type of treatment

Among the 229 consecutive patients between February 2018 and March 2019, we treated various diseases with common or rare conditions. However, several groups of diseases represented the largest portion of the patient’s problems. (Fig. 2) The majority of the patients suffered from traumatological and orthopedic diseases (34.7%). Within this group, most cases were wounds (44%), followed by older fractures, which may have been treated inadequately (13.6%). We observed the highest readmission rate (52%) in patients suffering from wounds compared to all other medical conditions. Infections (23.2%) presented the second most prominent cause for visits during consultation hours. About, 52.8% of the patients had an infection of the respiratory tract followed by urinary infections with 9.1%. The third group was treated for dermatological diseases (20.5%) with a primary focus on scabies (23.1%) and mycosis (15.5%). Significant differences in the distribution of diseases were found in regard to the gender of the patients. We observed a significantly higher proportion of dermatological diseases in male patients compared to females (*P* = 0.046). However, female patients suffered more often from endocrinological diseases (*P* = 0.035). No significant association was found between the type of disease and the country of origin of the patient (*P* = 0.933).

## Discussion

### What is already known on this topic

Student-Run Free Clinics have been essential institutions in US medical schools for a long time and have become popular institutions for students and patients in Europe in recent years. Only a few studies shared their experiences with patients of SRFCs in North America, but no study about the data of patients in European SRFCs is available.

### Main findings

This study describes the establishment of the second German SRFC in Hamburg and focuses on the countries of origin and disease entities. In the SRFC Hamburg, 229 patients underwent medical treatment between February 2018 and March 2019 with a readmission rate of 37.7%. The readmission rate resulted mostly from the chronic and complicated courses of the patients diseases with a necessity of a repetition of the treatment. Otherwise, a significant proportion of the patients welcomed the possibility of medical treatment after a prolonged time without access to medical care. A report from Germany revealed that around 65% of the homeless people had not accessed any healthcare service in the 6 past months at time of data collection.^27–29^^,^^32^ We observed that the majority of our patients had not been in contact with healthcare professionals for months or even years. A few patients were treated either in other institutions for underserved people or in hospitals due to emergency cases but not regularly by primary care physicians. Therefore, a number of patients lack access to regular medical care within a healthcare system with universal health coverage.^34^^,^^35^

Our patients reported various reasons why they used the SRFC medical service. The majority of our patients had a loss of insurance cover because they were not able to pay the monthly contributions due to their homelessness. Many patients had no valid residence permit and therefore no health insurance. Only a small portion of our patients was medically insured, but mostly avoided the contact to regular medical care due to their inadequate hygiene condition. In addition, some of these patients deny the opportunity of regular medical support. In summary, uninsured as well as insured patients do not access regular medical care even though the German healthcare systems offers universal health coverage for most acute conditions.^34^^,^^35^

Another key point of our study was to evaluate the countries of origin of our patients. Patients came from 33 different countries, which was a challenge for proper communication between medical students, health care professionals and the patient. One-third of the patients from within the European Union came from Eastern European countries, with very limited German or English language skills. For these patients, we created a network of students and professionals with a multilingual team on-call. With this team, we were able to establish appropriate communication with our international patients regardless of their language skills. When comparing our data with those from SRFCs in the USA, the language barrier seems to be more relevant in Europe. Only 58% of the US SRFCs have a language interpreter.^1^

Our study also represents the most common reasons for visiting our SRFC in Hamburg. Nearly 80% of the patients suffered from infections, traumatological, orthopedic or dermatological diseases. Treatment of acute or chronic wounds was the most common. These findings were different from the experience of US SRFCs, where the most common diagnoses were diabetes and hypertension. Those diseases played a secondary role in our SRFC in Hamburg.^1^ In contrast, our findings in Hamburg were in line with Canadian SRFCs. Ng *et al*. reported pain and infection as the most common reasons for visiting Canadian SRFCs, similar to our observations.^2^ When comparing our data with those of other specialized practices for homeless people from major cities in Germany, the most common diagnoses coincides to a large extent.^27^^,^^29^^,^^32^^,^^33^ The treatment of dermatological, infectious and parasitic diseases takes the most important place. In addition, Meidl *et al*. from Hanover reported about diseases of the circulatory system as one of their most common diagnoses.^27^^,^^32^ Furthermore, other studies from Berlin and North Rhine-Westphalia treated nearly 20% of the patients with mental and behavioral disorders, which is underrepresented in our study population.^27^ In contrast, the treatment of traumatological and orthopedic diseases is more relevant in our SRFC compared to the mentioned practices. In summary, we observed a large difference in the most common diagnoses between the US SRFCs and Canadian and German SRFCs.

This may be the result of the different health care systems in these countries and subsequent access to medication.

Our results demonstrate that patients suffering from wounds and old fractures are more likely to be treated multiple times. One reason is the poor compliance of most patients and an irregular appearance for treatments. Therefore, it was challenging for us to prevent chronic courses of the illnesses. Nevertheless, we offered professional patient care with our team of an experienced surgeon and a trained wound expert to reduce the readmission rate for these diseases. A similar issue was skin infection with scabies because of poor hygiene. In our experience, the interprofessional collaboration of SRFCs and homeless practices with shelters who can provide clothes and sanitary facilities for the patients is the only way to address this problem and to stop the worsening of the infection.^36^^,^^37^

Even though the primary objective of our SRFC is offering health care for a marginalized population, the volunteering students are also to benefit as they improve clinical knowledge and social accountability. Around 50 medical students were participating in our SRFC during the first year, where they either worked in consultation hours or managed the work processes of the SRFC. Many studies reported about a heightened social awareness and an increased interest to work with underserved after volunteering in a SRFC.^17–20^ In our particular case, we experienced that the majority of our students kept in contact with our SRFC after their graduation and showed willingness to volunteer in consultation hours further on.

In summary, our report highlights the importance and necessity of a SRFC in a major city in Northern Europe. Despite almost universal health coverage in Germany, a considerable number of individuals have limited access to health care and are dependent on alternative possibilities of medical treatment such as our SRFC. We therefore propose the need for medical treatment by SRFCs even in apparently functioning health care systems of developed countries.

### What this study adds

Our study reports for the first time about patients data of a European SRFC. The only available data come from Canadian and US SRFCs. It is worth asking whether how people can lack access to medical care in a country with universal coverage of health care, such as Germany. Our analysis clearly shows the need for medical treatment by SRFCs or similar services even in such apparently functioning health care systems. Furthermore, the use of SRFCs is a valuable instrument for improvement of the students’ ethical standards, professional and personal qualities.

### Limitations of this study

Since data from other European SRFCs are lacking, the possible variation of diseases and patients characteristics in different major cities needs further studies.

## Notes

**R. Drexler**, Research Associate

**F. Fröschle**, Master of Arts

**C. Predel**, Student

**B. Sturm**, Student, Bachelor of Arts

**K. Ustorf**, Student

**L. Lehner**, M.D.

**J. Janzen**, M.D.

**L. Valentin**, M.D.

**T. Scheer**, M.D.

**F. Lehnert**, M.D.

**R. Tadzic**, M.D. (Doc. Dr sc.)

**K.J. Oldhafer**, Professor, M.D.

**T.N. Meyer**, Professor, M.D.
